# Physiotherapy and pelvic floor health within a contemporary biopsychosocial model of care: From research to education and clinical practice

**DOI:** 10.4102/sajp.v77i1.1538

**Published:** 2021-05-20

**Authors:** Corlia Brandt

**Affiliations:** 1 Department of Physiotherapy, Faculty of Health Sciences, University of the Witwatersrand, Johannesburg, South Africa

**Keywords:** pelvic health, biopsychosocial model, evidence, research, women’s health, men’s health

## Abstract

**Background:**

Pelvic floor dysfunction (PFD) is a common problem in both men and women. Despite the high prevalence and negative effects on quality of life, there is still a lack of research in this area which translates into clinical practice and education.

**Objectives:**

My study discusses how gaps and controversies in current research and evidence on PFD might be addressed by positioning PFD within a contemporary biopsychosocial model of care (BPSM).

**Method:**

Various databases were searched for relevant studies published between 2010 and 2020 to support hypotheses and statements.

**Results:**

My study focuses on the available evidence of PFD in both men and women as related to the themes and sub-themes of the BPSM, and how this available evidence might translate into education and clinical practice. It highlights areas of research, education and clinical practice that need to be explored and how the different components of healthcare may influence one another.

**Conclusion:**

Biomedical aspects regarding pelvic health are mostly investigated and taught, whilst psychological, cognitive, behavioural, social and occupational factors, individualised care, communication and therapeutic alliances are still under-investigated and not integrated or translated at a sufficient level into research, education and clinical practice.

**Clinical implications:**

Incorporating the integration of all factors of the BPSM into research is important for effective knowledge translation and enhancement of a de-compartmentalised approach to management. The interaction between the different components of the BPSM should be investigated especially in a South African population.

## Introduction

Pelvic floor dysfunction (PFD) is a common problem experienced in both men and women. It includes problems such as urinary and faecal incontinence, pelvic pain, constipation, sexual dysfunction and pelvic organ prolapse – and the disorders often coexist (Bo 2012). Most studies report the estimates of prevalence of urinary incontinence in women to be between 25% and 45% (Dumoulin, Cacciari & Hay-Smith 2018) whilst up to 80% of men may experience urinary incontinence post-prostatectomy (Hodges et al. 2019). Prevalence of pelvic organ prolapse has been reported to be between 40% and 60% in parous women (Hagen & Stark 2011). Chronic constipation in the community may vary between 11% and 18% (Rao & Patcharatrakul 2016), sexual dysfunction in the general female population between 30% and 49% (Ferreira et al. 2015) and erectile dysfunction between 50% and 70% in men aged between 50 and 70 years (Harvard Men’s Health Watch 2020).

Despite the high prevalence of PFD, there is still a lack of research in this area and a lack of evidence regarding the most optimal management of many of these conditions. Healthcare and research, including the management of pelvic dysfunction, were originally based on a biomedical approach which focused only on biological factors, such as weak pelvic floor muscles (PFMs) in the case of PFD. Over the past decades, an alternative conceptual framework, namely, the biopsychosocial model (BPSM) was developed to help clinicians understand the interrelationship between biological, psychological and social aspects when approaching and addressing impairments in patients holistically. However, research, education and training and healthcare structures still sometimes reflect compartmentalised approaches when conceptualising the BPSM (Daluiso-King & Hebron 2020). Malalignment of evidence, teaching and training, clinical practice and research will unavoidably lead to suboptimal clinical outcomes and patient-centred care if not captured within a specified, integrated framework.

A recent review by Berghmans, Seleme and Bernards (2020) on the assessment of urinary incontinence emphasises many of the above-mentioned BPSM factors as important to identify any local or general impeding or prognostic factors that can affect the recovery or adjustment processes in patients with PFD. It is important to assess patients’ movement patterns, respiration, general physical and psychological status, illness beliefs, consequences for aspects of everyday life such as work, sports participation, housekeeping, sexuality, social and family life, and contextual factors (external and personal) such as motivation and adherence (Berghmans et al. 2020). The question, however, remains whether compartmentalisation still exists and how these factors are incorporated and viewed in current research topics, teaching and training, and clinical practice to improve the understanding, research and implementation of a holistic BPSM as applicable to patients with PFD.

The BPSM also relates closely to the International Classification of Function, Disability and Health (ICF) which describes PFD in terms of impairment (closely related to the biomedical aspect of the BPSM), disability and restriction of participation (relating to the master themes and sub-themes of the BPSM). Impairments refer to the abnormality or loss of physiological, psychological or anatomical structures or function at the level of the organ (WHO 2018). In the context of PFD, this refers to impairments such as detrusor overactivity, stress urinary incontinence, over- or underactive PFM, tears in the PFM or connective tissue and erectile dysfunction. Disability refers to problems such as straining with defaecation or involuntary loss of urine during activities as it describes the loss or restriction of the person’s ability to perform functions or activities in a normal manner. These limitations and restrictions usually lead to the disability that prevents or limits the fulfilment of a normal role in society. This may include the role as a spouse (sexual dysfunction), mother (cannot play with children without leaking) or provider (cannot go to shops to purchase food or work), and not being able to attend social, work or sports activities amongst many others. However, these dysfunctions are also closely related to contextual (external and personal) factors such as age, gender, not being able to attend visits to health practitioners, poor adherence to management programmes and lack of motivation (Berghmans et al. 2020; Brandt & Van Vuuren 2019; WHO 2018). Within these frameworks, reasons should, therefore, be sought for the current gaps and controversies that are still found in research and training on pelvic health.

Urinary incontinence in women is probably the most researched area with clear evidence on the efficacy of pelvic floor muscles training (PFMT) (Dumoulin et al. 2018). Questions still remain regarding the effectiveness of treatment in patients with sexual dysfunction and pelvic organ prolapse (Bo 2012). Some clinical trials on the effectiveness of treatment for urinary incontinence post-prostatectomy have shown positive outcomes, but a meta-analysis from a systematic review could not show evidence of efficacy (Anderson et al. 2012). This lack of overall estimates confirming efficacy seems to apply to the other areas of PFD as well.

Unfortunately, this uncertainty translates into education and clinical practice. There is still limited training on pelvic and women’s health at both under- and post-graduate levels when compared with other areas of physiotherapy (Frawley, Neumann & Delany 2018). This might also explain the limited number of therapists who practice in this field when compared to other areas. Medical aids are also reluctant to cover costs for PFD treatment in both women and men because of the limited evidence on efficacy. The development of research and evidence on PFD is, therefore, crucial to enhance education, training and clinical practice to address the need for care as indicated by the high prevalence rates of PFD in society.

My study aimed to discuss the evidence of PFD within a contemporary BPSM and the implications for research, education and training and clinical practice.

## Materials and methods

A literature search was performed for studies published between 2010 and July 2020. The following databases were searched: PubMed, Medline, Embase, CINAHL, PEDro and the Cochrane database. Publications focusing on biomedical, psychological and social aspects, communication and individualised care in patients with PFD were retrieved. These included systematic reviews, randomised controlled trials and observational study designs. The reference lists of these studies were also checked for any applicable literature.

Information from the studies was extracted and summarised in Table 1, which were applicable and amended for the purpose of my study. The number of studies covering each of the above aspects is also indicated in Table 1.

**TABLE 1:** Themes and sub-themes of the biopsychosocial model of care investigated by the studies reviewed.

Study	Study design	BPSM themes								
		Biomedical	Educational	Psychological	Cognitive	Behavioural	Social	Therapeutic alliance		Individualised care
								Occupational	Communication	
Aljuraifani et al. (2019)	Cross-sectional (healthy women)	PFM activity	-	-	-	-		-	Verbal instructions	-
Brennand et al. (2018)	Descriptive study on urinary incontinence in women	-	Awareness of treatment	-	-	Adaptive behaviours	Physical activities	-	-	-
Dumoulin et al. (2018)	Systematic review on treatment of urinary incontinence in women	Symptomatic cure, PFM function, general health	-	-	-	Patient adherence	Sexual dysfunction	-	-	-
Woodley et al. (2020)	Systematic review on treatment of urinary and faecal incontinence	Symptomatic cure, PFM function	-	-	-	-	Quality of life	-	-	-
Hagen and Stark (2011)	Systematic review on treatment of pelvic organ prolapse in women	Symptomatic cure, PFM function	-	-	-	-	Quality of life	-	-	-
Imamura, Wells and McGrother (2015)	Systematic review on the effect of lifestyle interventions on urinary incontinence in men and women	Symptomatic cure	-	-	-	Lifestyle	-	-	-	-
Candy et al. (2016)	Systematic review on interventions for sexual dysfunction	Symptomatic cure	-	Psychotherapy/psychoeducational	-	-	Sexual dysfunction	-	-	-
Preda and Moreira (2019)	Literature review	Symptomatic cure, PFM function	-	-	-	-	Sexual dysfunction			
Rajkowska-Labon et al. (2014)	Randomised controlled trial (RCT) on the effect of PFMT post-prostatectomy	Symptomatic cure, PFM function	-	-	-	-	-	-	-	-
Dorey et al. (2004)	RCT on the effect of PFMT on erectile dysfunction	Symptomatic cure, PFM function	-	-	-	-	Quality of life			
Milios, Ackland and Green (2019)	RCT on the effect of PFMT on urinary incontinence	Symptomatic cure, PFM function	-	-	-	-	Quality of life	-	-	-
Pastore et al. (2018)	Retrospective study on the effect of PFMT on premature ejaculation	Symptomatic cure	-	-	-	-	-	-	-	-
Ferreira et al. (2015)	Systematic review on the effect of PFMT on sexual function	Symptomatic cure, PFM function	-	-	-	-	Sexual dysfunction the quality of life	-	-	-

BPSM, biopsychosocial model of care; PFMs, pelvic floor muscles; PFMT, pelvic floor muscle training.

## Current evidence and research focus areas as related to the BPSM

Research in the field of pelvic and women’s health physiotherapy originally focused mainly on the biomedical aspects (impairments) of PFD. This phenomenon might partially be explained by the literature emphasising that movement impairment should provide the basis for diagnosis and treatment of dysfunction (Spitznagle et al. 2017). This statement is correctly based on the signature pedagogy of physiotherapy that is defined as ‘human movement’ (Jensen et al. 2017). However, it is also stated that diagnosis and treatment should be guided by a combination of symptoms, signs and impairments related to movement, activity and participation restrictions which might require a deeper understanding and investigation within the BPSM (Spitznagle et al. 2017).

Daluiso-King and Hebron (2020) conducted a concept analysis and expansion on the current BPSM. Their findings propose a holistic framework for musculoskeletal care. The framework includes five master themes and five sub-themes under which PFD will be further discussed (Table 2).

**TABLE 2:** Master and sub-themes of the biopsychosocial model of care.

Master themes	Sub-themes
Biomedical (clinical reasoning, subjective assessment, pain, treatment, assessment)	Education (re-conceptualising beliefs, health promotion, stay active advice, pain education)
Psychological	Cognitive (perceptions, illness beliefs, beliefs, self-efficacy, expectations) and behavioural (catastrophising, coping strategies, fear avoidance). Behavioural change includes readiness, motivation, attitude, patient engagement. It also includes feelings and emotions (anxiety, depression, fear).
Social (support systems, environmental and cultural factors, legal compensations)	Occupational (socio-economic, unemployment, ergonomics, job satisfaction)
Communication (motivational interviewing, counselling problems and solving skills, listening, patient preferences, reflective approach, language aspects, reassurance, understanding and knowing the patient)	Therapeutic alliance
Individualised care (integrated approach, holistic care)	

*Source*: Daluiso-King, G. & Hebron, C., 2020, ‘Is the biopsychosocial model in musculoskeletal physiotherapy adequate? An evolutionary concept analysis’, *Physiotherapy Theory and Practice* 16, 1–17. https://doi.org/10.1080/09593985.2020.1765440

### Biomedical and educational factors

Research in this domain has mainly focused on the effects of PFMT on the symptoms of incontinence, pain or symptoms of underactive or overactive PFMs, the morphological characteristics and functional anatomy of specifically the pelvis and its surrounding muscles underlying the impairments. The sub-theme of education is occasionally incorporated into the studies on effectiveness, reporting on the outcomes of home exercise programmes and advice on lifestyle (Dumoulin et al. 2018; Hagen & Stark 2011; Milios et al. 2019; Nahon et al. 2006; Rajkowska-Labon et al. 2014). However, aspects such as re-conceptualisation of patient beliefs and health promotion are rarely investigated or reported on, as will be discussed below.

A recent systematic review by Dumoulin et al. (2018) concluded that PFMT can improve and/or cure symptoms of urinary incontinence in women. The studies included in the review focused mainly on biomedical measures or impairments as primary outcome measures, namely, symptomatic cure. The findings indicate that only three trials reported on patient’s satisfaction, one on self-efficacy, two on general health status measures and one on sexual measures, whilst 36 trials reported on objective measures of PFM function as secondary measures. This demonstrates a tendency towards the assessment of biomedical factors.

Some evidence is available on the beneficial effect of PFMT on prolapse symptoms and severity in women related to outcomes on anatomical position and symptomatic improvement (Hagen & Stark 2011). However, this systematic review by Hagen and Stark (2011) clearly states the need for other comparisons in trials on pelvic organ prolapse, the effects of lifestyle interventions and trials investigating prolapse prevention. Of the four included studies, only two reported on patient satisfaction and perception, whilst no data were reported by the studies on the generic quality of life, sexual function, economic measures or psychological outcomes.

Evidence on the efficacy of PFMT for urinary incontinence post-prostatectomy is scarce when compared with the abundance of literature available for PFD in a female population. A review by Nahon et al. (2006) concluded that well-designed studies and randomised clinical trials are needed to clarify the most effective treatment protocols and components to improve post-prostatectomy urinary incontinence in men. A more recent randomised clinical trial investigated the effect of PFMT on continence symptoms in 81 men following radical prostate only prostatectomy. They found a significant improvement in continence symptoms in the intervention group, but no outcomes on any other aspects of the BPSM were noted (Rajkowska-Labon et al. 2014). A more recent randomised clinical trial by Milios et al. (2019) demonstrated improved outcomes in incontinence, quality of life and PFM function in men who underwent radical prostatectomy after receiving peri-operative physiotherapy.

Hodges et al. (2020) recently published a study where they stated that outcomes of PFMT programmes to treat and prevent incontinence after radical prostatectomy are inconsistent. He proposes targeted programmes tailored to the individual patient’s needs, also considering additional factors such as management of bowel dysfunction, maintenance of mental health, reinforcement of general exercise and weight loss and advice regarding erectile dysfunction and penile rehabilitation. This viewpoint relates to the suggested BPSM and inclusion of the psychological, social and individualised care themes and sub-themes. No evidence is available yet on this approach, but studies have been undertaken and their results may be interesting.

### Psychological, cognitive and behavioural factors

I have noticed that the focus in research has started to shift towards the investigation of psychological aspects (including both cognitive and behavioural issues) in patients with PFD and how these are integrated into the dysfunction. Aspects such as feelings and emotions are mainly covered in quality-of-life assessments; whilst other aspects including perceptions, illness beliefs, general beliefs, self-efficacy, expectations, catastrophising, coping strategies, fear avoidance, readiness, motivation, attitude and patient engagement are still under-investigated whilst looking at the focus of primary outcomes in the majority of studies. However, there are emerging studies for investigating these factors, as indicated in the following paragraphs.

Brennand et al. (2018) conducted a survey in a cohort of physically active women to investigate the psychological and social factors related to physical activities that lead to urine loss, the consequent coping mechanisms and the awareness and interest of women to seek help for the dysfunction. They found that it was mostly high-impact physical activities that were affected and that women commonly demonstrated adaptive behaviour. However, of considerable importance for clinical practice and from a patient education perspective is that the women indicated the interest to seek treatment of the dysfunction might reflect on their motivation, illness beliefs and aspects such as behavioural change.

A systematic review by Imamura et al. (2015) included 11 trials on the effects of lifestyle interventions on urinary incontinence in both men and women. They only found studies investigating the effect of weight loss, type of diet, volume of fluid intake and caffeine intake. No studies were found that investigated the known risks of the development of incontinence such as avoiding constipation, alcohol intake, stopping smoking, straining or physical activity levels. They concluded that there is insufficient evidence to inform clinical practice on the beneficial effect of the included lifestyle interventions. They did, however, find an increase in evidence on the beneficial effect of weight loss in overweight women to reduce urinary incontinence and, therefore, proposed this as a priority area for future research.

### Social and occupational factors

Social and occupational aspects have rarely been investigated in patients with PFD. This is concerning as the main outcome of the management of the patient should be the fulfilment of his or her normal role in society according to the ICF (WHO 2018). The only evidence related to social factors and PFM function is the studies investigating sexual dysfunction which relates to their social role as a spouse (Berghmans et al. 2020). It is, however, important to remember that sexual dysfunction cannot be classified solely under any aspect of the BPSM, as it includes a complex interaction of factors overlapping all aspects of the BPSM. It can be interpreted as either a biomedical aspect leading to social dysfunction or a social factor affecting other aspects of the BPSM. This is reflected in the definition of sexual dysfunction which includes psychological changes, a disturbance in sexual desire, changes in sexual response, interpersonal difficulty and distress, effects on lifestyle and self-esteem. Risk factors include relationships with partners, menopausal status, pregnancy, general health conditions, alcohol consumption and nicotine use (Ferreira et al. 2015). The purpose of the below referenced studies is, therefore, to discuss the compartmentalisation when investigating sexual dysfunction from a physiotherapeutic perspective.

Evidence is emerging regarding PFMT and PFM function as related to sexual dysfunction (Candy et al. 2016; Ferreira et al. 2015; Pastore et al. 2018; Preda & Moreira 2019). This tendency is evident in the publication of protocols and reviews such as by Sobhgol et al. (2019) planning to investigate the effect of ante-natal PFMT on female sexual function during pregnancy and 3 months following delivery.

A previous systematic review by Candy et al. (2016) reviewed the effectiveness of interventions for sexual dysfunction following the treatment of cancer in women. Only one study was identified focusing on the PFMs, exercises and the effect on the dysfunction. Only symptomatic cure and mixed outcomes for sexual function were reported. A similar tendency in outcome measures was seen in a systematic review by Ferreira et al. (2015) on the effect of PFMT for sexual dysfunction. Most studies included in their review found at least one aspect of sexual dysfunction that improved with PFMT, but these aspects differed amongst studies. They concluded that the topic remains understudied and that more randomised controlled trials are needed.

More recently, a review by Preda and Moreira (2019) identified 12 publications that investigated the effect of PFM rehabilitation on sexual function in women with stress urinary incontinence. They concluded that PFM rehabilitation can improve sexual dysfunction in this population because of decreased episodes of incontinence as well as improved PFM strength.

Sexual dysfunction in men is mostly experienced post-prostatectomy because of erectile dysfunction or in the form of premature ejaculation. Evidence is still scarce on these topics and they need further investigation. A small randomised clinical trial has indicated improved outcomes after a 3-month PFMT programme on measures such as quality of life and symptomatic outcomes related to erectile dysfunction (Dorey et al. 2004).

Pastore et al. (2018) conducted a retrospective review on 122 patients with lifelong premature ejaculation and found a significant long-term improvement in patients who received PFMT. All the patients had other unsuccessful previous treatments which did not include PFMT. Limitations of this study would be that it was retrospectively conducted and that no control group was included. Clinical trials are, therefore, still needed to investigate the efficacy of such PFMT regimes.

A recent Cochrane review by Philippou et al. (2018) investigating the effectiveness of penile rehabilitation on erectile dysfunction did, however, not include physiotherapy as a method of intervention. The reason could be the lack of literature and evidence in physiotherapy on this topic. Wong, Louie and Beach (2020) confirmed this in their review on the effectiveness of PFMT for erectile dysfunction post-prostatectomy, stating that there is limited high-quality evidence which led to drawing few conclusions. Future research should focus on the strategies to improve patient adherence, clearly describe exercise protocols and integrate evidence on communication such as verbal cues and biofeedback into their methodology.

It is, therefore, clear from the above reviews, the complex definition of sexual dysfunction and the integrated aspects of the BPSM, that compartmentalisation still exist when the topic of sexual dysfunction is investigated.

### Individualised care, communication and therapeutic alliance

Individualised care and communication are closely linked to create a therapeutic bond which facilitates a deeper understanding of the patient. This is achieved by means of aligning and sharing of the therapist’s and patient’s beliefs, thoughts and emotions (Daluiso-King & Hebron 2020). These concepts are, however, not addressed or considered in high-quality research interventions, as they are usually seen as a form of bias and confounding factors which will interfere with the standardised execution of protocols. The question should, however, be asked if this approach is not actually preventing us from investigating the most applicable or effective holistic approach as suggested by models such as the BPSM. Individualised care is, however, being introduced in intervention studies in the form of investigating patient-specific exercise programmes (Berghmans et al. 2020).

A similar argument can perhaps be made why communication has also received less attention in studies on PFD. However, good evidence exists on the efficacy of good communication skills to improve the therapeutic alliance in neuromusculoskeletal therapy (Pinto et al. 2012), which is also the umbrella under which PFMT and PFD lie. Information can, therefore, be drawn from these studies to guide further research in pelvic health.

Aljuraifani et al. (2019) investigated the effect of different verbal cues on the contraction of the superficial and deep PFM with electromyography in a cross-sectional study design. They found that healthy women activated their superficial PFMs to a greater proportion when compared with the deep PFMs across all instructions, whilst specific instructions led to the best activation of the superficial PFMs. Their study, therefore, emphasised that the type of verbal instructions that we use can influence the pattern of activity of the PFMs.

### Recommendations for future research

Although it seems that research related to PFD is evolving as related to a contemporary BPSM, the focus and outcomes of studies are still compartmentalised in most instances instead of conceptualising it as a holistic approach and investigating interactions, as depicted in Table 1. Primary outcomes are isolated and rarely interactions between the biopsychosocial aspects are determined. There are a couple of postulated reasons that can be considered.

Researchers try to limit environmental, personal and social factors in studies (unless these are the topic of investigation), as they are seen as confounding factors that would compromise the validity of the results. The question should, however, be asked to what extent does this approach compromise the validity and generalisability of research findings and the investigation of the holistic treatment approach, where the overlap of BPSM themes and removing of boundaries amongst themes are crucial for the integration of concepts (Daluiso-King & Hebron 2020). This suggestion is supported to an extent by the conclusion of Woodley et al. (2020) in a systematic review on the prevention and treatment of urinary and faecal incontinence in antenatal and post-natal women. The authors postulate that the treatment and prevention effects might be more effective with targeted rather than mixed approaches, which should be specific for certain populations. They suggest that physiological and behavioural aspects of exercise programmes should also be described and incorporated.

Ferreira et al. (2015) also support the above postulation by suggesting that when investigating sexual function or dysfunction in patient populations, future studies should consider including variables such as contextual, interpersonal, personal, biological and psychological factors in their research hypotheses and questions. The results of studies should then also consider the effect of PFMT on sexual function in the context of these variables.

## Research, clinical practice and education

Unfortunately, compartmentalised investigations and outcome measures as well as a lack of research on aspects of the BPSM are translated into clinical practice and training where challenges arise in the understanding and holistic treatment of patients.

Clinical competence can be defined as the use of technical skills, knowledge, communication, clinical reasoning, values, emotions and reflection to be used to the benefit of the patient or community (Frawley et al. 2018). Although we have good knowledge on biomedical aspects, we still need to fill the gaps regarding more research in the other areas such as communication, cognitive, emotional and educational topics on PFD from both a patient and clinician perspective.

One of the most important predictors of effective healthcare, implementation of research knowledge, knowledge transfer and change in healthcare is amongst other aspects successful communication: as related to patient–clinician interaction as well as between researchers and healthcare practitioners (Pierce et al. 2015). Little evidence, however, exists on this communication topic as related to BPSM and PFD and patient–clinician interactions. Other communication issues include some discrepancy regarding the use of terminology such as ‘pelvic floor disorders’, ‘pelvic floor dysfunctions’ and ‘pelvic health’ amongst healthcare professionals, researchers and the wider public.

Good communication is, therefore, very important, not only for patient assessment but also for the transfer of appropriate knowledge. An example is where the World Health Organization (WHO 2018) defines health as ‘a state of complete physical, mental and social well-being and not merely the absence of disease or infirmity’. This definition has, however, not been translated correctly into the context of PFD. Correct understanding and communication of this definition, therefore, defines pelvic floor health as a concept that encompasses physiological and anatomical integrity of the pelvic floor structures and the effect that PFD has on an individual’s psychological and emotional state within a specific social context. Pelvic floor health will also, therefore, depend on life events and predispositions such as internal factors (ageing and genetic makeup) and external factors (culture, socio-economic situation, lifestyle and life events). Optimal pelvic floor health through life with the prevention of disorders, disease and dysfunction is, therefore, a multifactorial and complex process as also indicated by the BPSM in the previous sections. A clear understanding, investigation, communication and application of concepts as related to pelvic floor health is, therefore, a priority amongst professionals and researchers (Pierce et al. 2015).

Pierce et al. (2015) proposed the following definition of ‘pelvic floor health’ as established by a concept analysis:

Pelvic floor health refers to the physical and functional integrity of the pelvic floor unit through the life stages of an individual (male or female), permitting optimal quality of life through its multifunctional role and where the individual possesses or has access to knowledge, which empowers the ability to prevent or manage dysfunction. (p. 999)

Indicated by the prior literature discussion of the BPSM concepts and supported by the above concept analysis, clinicians, educators and researchers should incorporate this comprehensive definition of pelvic floor health in their practice and guidelines. Although the biomedical aspect is an important concept to understand as it underpins the impairments related to the neuromusculoskeletal system in patients with PFD, attention should be given to the interrelationship with other factors of the BPSM to comprehend and research the holistic approach as is encouraged and taught in theory. Although some studies include data on social and psychological themes, these data are not used to compile patient-centred treatment protocols for studies or when treating patients. As indicated, most studies include quality-of-life assessments as a subjective measure to determine symptomatic cure, psychological and social aspects but do not determine the relationship or interaction with other factors of the BPSM and how to incorporate all factors into the management of the patient.

In conclusion, linking clinical practice, research, education and knowledge translation, as well as the very complex interactions there can be amongst aspects of the BPSM other than the biomedical, can be explained by the following example on the management approach to PFD. Correct training of a patient requires cognitive skills and understanding from the patient and an effective exercise prescription from the therapist. The patient, therefore, can be seen as an adult learner in this process, which in turn requires additional skills and understanding from the therapist regarding adult learning to deliver the information effectively. One of the guiding principles for effective learning to take place is to consider the patient’s life stages and life events. The learning environment of the patient should reflect the physical, affective-social and intellectual dimensions needed to create an optimal learning climate (Plack & Driscoll 2011; Smith 2009). Following a biopsychosocial approach and taking into account the creation of an optimal learning environment emphasise the need to determine and understand all aspects of the BPSM (as related to the patient and therapist) to compile an individualised training programme for the patient. The latter plan of care optimises patient adherence but at the same time demonstrates the complexity of clinician and patient interaction and communication to lead to effective treatment (Kurtz et al. 2003).

## Conclusion

Research reviewed in this manuscript, as related to the contemporary BPSM and PFD in men and women, has mainly focused on the investigation of biomedical aspects. Evidence exists on the beneficial effects of PFMT for PFD such as incontinence, pelvic organ prolapse (POP) and sexual dysfunction. Psychological, cognitive, behavioural, social and occupational factors, individualised care, communication and therapeutic alliances are still under-investigated from a physiotherapeutic perspective. The research related to PFD in men is also still lacking when compared with the evidence available on PFD in women. Incorporating the integration of all factors of the BPSM (themes and sub-themes) into research is important for effective knowledge translation and enhancement of a de-compartmentalised approach into training and clinical practice. Future studies should, therefore, focus on research topics other than biomedical ones or incorporate outcome measures in efficacy studies as related to all aspects of the BPSM. It is also important that the interaction between the different aspects of the BPSM as related to PFD be determined in the future studies.

## Limitations

The recommendations and postulations in this study should further be investigated by means of a systematic review to come to a definite conclusion on this topic.
